# Hierarchical structure in the world’s largest high-speed rail network

**DOI:** 10.1371/journal.pone.0211052

**Published:** 2019-02-13

**Authors:** Sheng Wei, Shuqing N. Teng, Hui-Jia Li, Jiangang Xu, Haitao Ma, Xia-li Luan, Xuejiao Yang, Da Shen, Maosong Liu, Zheng Y. X. Huang, Chi Xu

**Affiliations:** 1 School of Architecture and Urban Planning, Nanjing University, Nanjing, China; 2 School of Life Sciences, Nanjing University, Nanjing, China; 3 Jiangsu Institute of Urban Planning and Design, Nanjing, China; 4 School of Management Science and Engineering, Central University of Finance and Economics, Beijing, China; 5 Institute of Geographic Sciences and Natural Resources Research, Chinese Academy of Sciences, Beijing, China; 6 Nanjing Puhou Ecological Technology Company Limited, Nanjing, China; 7 College of Life Sciences, Nanjing Normal University, Nanjing, China; University of Sussex, UNITED KINGDOM

## Abstract

Presently, China has the largest high-speed rail (HSR) system in the world. However, our understanding of the network structure of the world’s largest HSR system remains largely incomplete due to the limited data available. In this study, a publicly available data source, namely, information from a ticketing website, was used to collect an exhaustive dataset on the stations and routes within the Chinese HSR system. The dataset included all 704 HSR stations that had been built as of June, 2016. A classical set of frequently used metrics based on complex network theory were analyzed, including degree centrality, betweenness centrality, and closeness centrality. The frequency distributions of all three metrics demonstrated highly consistent bimodal-like patterns, suggesting that the Chinese HSR network consists of two distinct regimes. The results indicate that the Chinese HSR system has a hierarchical structure, rather than a scale-free structure as has been commonly observed. To the best of our knowledge, such a network structure has not been found in other railway systems, or in transportation systems in general. Follow-up studies are needed to reveal the formation mechanisms of this hierarchical network structure.

## Introduction

While the mobile trajectories of human beings can vary greatly from person to person, strong regularities in collective human mobile patterns often arise as fascinating emergent properties in social systems [1,2]. Many human mobile patterns are associated with public transportation systems (PTSs) [3,4]. In addition, travelling behaviors of humans strongly shape the structure and functions of PTSs [5]. The characterization of the structure, functions and dynamics of PTSs is a fundamental step to understanding human mobile patterns, as well as the influences that PTSs have on the surrounding physical and cultural landscapes [6–9].

System-wide characterizations of PTS structure and functioning are gaining momentum, boosted by theoretical advancements and big data. Recent progress in complex network science has shed light on this topic from the topological network structure point of view. From a complex network perspective, a system can be generalized as a network based on graph theory [10]. The basic representation of a network is a set of nodes (vertices) that represent system components, and a set of edges (links) that represent connections and interactions between nodes. While specific approaches for network representations of PTSs can be different across different studies (e.g., the L-space vs. P-space approaches, see Materials and methods), some interesting common features have been observed. A striking finding from complex network analyses is that various PTSs have very similar topological properties, giving rise to an exciting possibility that those distinct systems could be shaped by essentially the same underlying mechanisms and could share common dynamics, regardless of the PTS type or geographic region. Such commonalities can be reflected by the well-known scale-free [11,12] and small-world properties [13], which have been reported in many PTSs including railway [14,15], metro [16,17], maritime [18–20], and airplane [21] systems, as well as a number of network systems, such as power grids [22,23], social networks [24,25] and the Internet [26,27]. Although the suggestion that there may exist universal network properties across systems is indeed appealing, there are some noteworthy deviations and exceptions [28]. So far, complex network-based investigations of PTSs are still far from exhaustive, leaving much room for obtaining a fuller picture of their regularities and variabilities.

Systematic studies on PTSs have been largely restricted by data availability. With the rapid development of digital technologies, many ‘smart’ PTSs are now able to record passenger flows in real time. This facility makes it possible for the public to access relevant information on PTS operation, providing an opportunity to analyze system behaviors in depth with such big data. For example, the big data produced by automatic fare collection systems (e.g., data from smart cards) can be used to characterize passenger flows [29], and regularities and variabilities of statistical patterns of human mobility [30]. The potential of open big data is just beginning to be explored. For example, freely accessed information on remaining tickets from ticketing websites can be assimilated and synthesized for retrieving passenger flow and occupancy rates, and thereby depicting PTS functions [31]. This information is also valuable for characterizing the holistic spatial structure of PTS networks [32].

Railway systems play a critical role in intercity transportation. There has long been interest in the structure of railway systems. Previous studies on nationwide railway networks (e.g., Indian, Polish, Swiss, Japanese, and Chinese railway systems) have suggested that they have exponential or power-law-like degree distributions [14,15]. However, our understanding of system-wide patterns and behaviors remains largely incomplete, limited by data availability on their extensive numbers of stations and routes. High-speed rail systems (HSRs) are among the most efficient PTSs. Since the first ‘bullet trains’ were initiated in Japan in the 1960s and in Europe in the 1990s, HSRs have created important socioeconomic impacts in many ways at both the regional and national levels. The situation is especially conspicuous in China. In less than 15 years, China has built HSR routes exceeding 22,000 km, ranking first in the world, with their length still growing rapidly [33]. Despite their relatively short time period of existence, Chinese HSRs are expected to profoundly influence Chinese socio-geographical systems in many aspects [34–37]. However, so far, our understanding of the world’s largest HSR system remains limited (but see [31,32]).

Does this unique PTS present the commonly observed network properties as conventional railways systems, or does it have rather distinct features? Hampered by data inaccessibility, this question has been difficult to answer. In this study, we used a publicly available data source, namely, information from a ticketing website, to collect an exhaustive dataset on the stations and routes within the Chinese HSR system. We analyzed a set of classical metrics that measure the relative importance (in terms of centrality) of nodes in the network. We demonstrated that the world’s largest and fastest growing HSR system has a distinct network structure in contrast with previously studied railway systems, as well as many other PTSs.

## Materials and methods

### Data acquisition of the HSR network

Construction of the nationwide HSR system of China began in 2004. Currently, the Chinese HSR system covers approximately 22,000 km, which is 60% of the total distance covered by the high-speed railways in the world. This HSR system is estimated to cover 80% of major Chinese cities (*‘Mid- and Long-term Planning of Railway Networks in China’*). The maximum train speed of the Chinese HSR is above 350 km per hour, enabling passengers to travel across the major part of mainland China within half a day.

The Chinese railway ticketing website (www.12306.cn) contains information on all planned HSR trains, including their routes and stations (Hong Kong, Macau and Taiwan excluded). This website provides a set of application programming interfaces (APIs) that enable automatic searches for this information. In this study, a program written in the C# language and using this API was developed to obtain data on all HSR trains in June, 2016, involving 704 stations. This dataset was then used to represent the HSR network. Detailed information regarding data acquisition can be found in Wei et al [32].

### Network analysis

PTS networks have been frequently represented in L-space or P-space. In L-space, stops or stations are represented as vertices, and the link between a given vertex pair is realized if the vertices are ‘consecutive on an arbitrary route’ (also referred to as the space of stops or space of stations [38]). In contrast, the P-space approach (also referred to as the space of transfer [38]) establishes a link between nodes if there is at least one line connecting two nodes. In this work, the Chinese HSR system is described in P-space.

Three frequently used complex network metrics were used, including degree centrality, betweenness centrality, and closeness centrality. Passenger flow volume is probably the most meaningful variable for characterizing the weight of stations. However, exact flow volume data were not available in this dataset. Note that in our previous work [32], train frequency was used as a rough proxy of flow volume. However, as transportation capacity (number of total seats) and occupancy rate can vary highly between different trains, train frequency may be an inaccurate indicator of flow volume. As frequency distribution patterns of the metrics might be particularly sensitive to any such bias, we used non-weighted metrics.

#### Degree centrality

The simplest metric measuring centrality is degree centrality (Eq 1).

$$C_{d}(v)=deg(v)$$ (1)

where *C*_*d*_(*v*) is the degree centrality of a node *v*; deg(*v*) is the degree of node *v* (i.e., the number of edges incident upon node *v*). Note that the studied network was confirmed as being undirected.

#### Betweenness centrality

Betweenness centrality is used to measure the importance of a node as a necessary intermediate on the shortest paths between all node pairs across the network [39–41] (Eq 2):

$$C_{B}(v)=\sum_{s\neq v\neq t}\frac{\sigma_{st}(v)}{\sigma_{st}}$$ (2)

where *C*_*B*_(*v*) is the betweenness centrality of a node *v*; *σ*_*st*_ is the total number of shortest paths between node *s* and node *t*; and *σ*_*st*_(*v*) is the number of those paths that pass through node *v*.

#### Closeness centrality

Closeness centrality measures how close it is from a given node to all other nodes in a network (Eq 3):

$$C_{c}(v)=\frac{1}{\sum_{t}d(v,t)}$$ (3)

where *C*_*c*_(*v*) is the closeness centrality of a node *v*; *d*(*v*,*t*) is the distance (i.e., the number of edges in a shortest path) between node *v* and node *t*.

The three network metrics were calculated using the software Gephi 0.9.1. Their frequency distributions and probability density curves were plotted in Matlab 2011b with the *ksdensity* function. The nodes (stations) were projected on the geographical map using ArcGIS 10.1.

## Results and discussion

**Fig 1:**
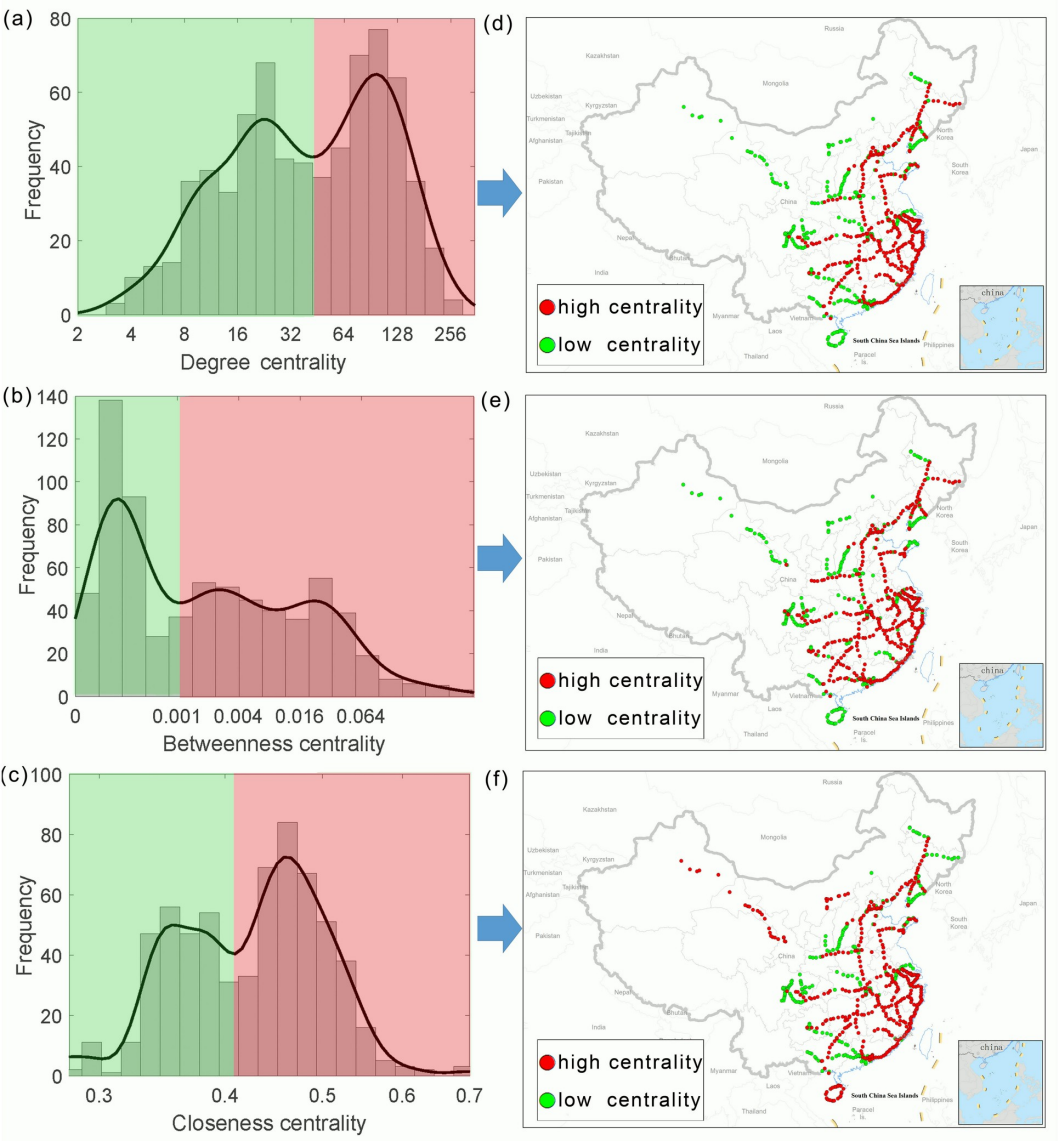
Frequency distributions of degree (a), betweenness (b), and closeness centrality (c) of the Chinese high-speed rail network. All metrics are plotted on a logarithmic scale. The system can be divided into two regimes (red vs. green) using a cut-off at the local minima of the probability density curves (**a-c**) calculated using *ksdensity* function in Matlab 2011b. The high- and low-centrality stations are placed on the map, as represented by red and green dots, respectively (**d-f**).

**Fig 2:**
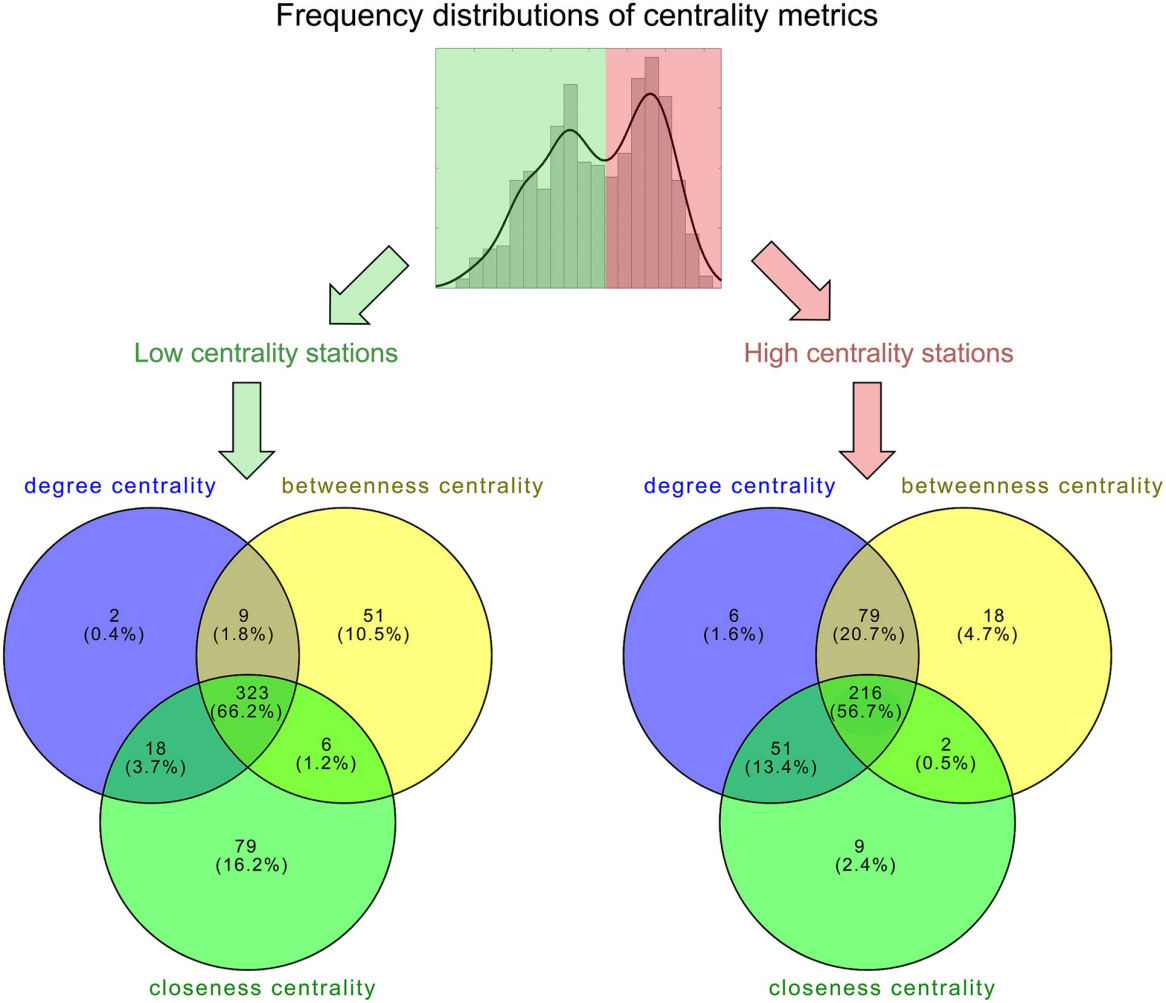
Venn diagrams for the high- and low-centrality stations identified for the three network metrics. Each colored circle representing a high- or low-centrality station set is identified from the frequency distribution of a given network metric (see Fig 1). Overlapping regions of circles indicate the number (proportion in the brackets) of the identical stations identified by corresponding metrics.

Our analyses of the four network metrics (degree centrality, betweenness centrality, and closeness centrality) demonstrated a striking structure of the Chinese HSR system. The frequency distributions of all three metrics presented consistent bimodal-like patterns (Fig 1a–1c). One possible explanation of such bimodal-like patterns is that the Chinese HSR system consists of two distinct regimes. For each metric, a cut-off was used at the local minima on its probability density curve to distinguish between low-centrality and high-centrality nodes (green vs. red parts in Fig 1a–1c). Such partitions gave rise to a consistent pattern across the three network metrics: both the low- and high-centrality parts consist of a largely similar set of stations across different metrics (Figs 1d–1f and 2). These results reinforce the suggestion that the bimodal-like distributions essentially reflect a two-regime structure in the Chinese HSR system.

A close scrutiny of individual metrics resulted in specific insight into the network structure. The observed bimodal degree distribution indicated that each of the two regimes in the network had a ‘characteristic scale’, or a ‘characteristic degree’, as indicated by the modes denoting the connection numbers that were most frequently found (i.e., the peaks in the degree distribution reflected typical scales in the network). Such a degree distribution is especially noteworthy, in the sense that it is quite different from the previously reported network properties of railway systems, as well as other PTSs. Exponential degree distributions have been repeatedly found in Indian [15], Polish, Swiss and Central European railway systems (in P-space) [38], while power-law degree distributions have been observed in Japanese systems (in L-space) [42], Chinese systems (in both L- and P-space [14, 43–44]) and Indian [45] railway systems. In addition, a similar power-law-like property has also been observed in airline networks, which are also important PTSs for intercity travel [21]. However, in contrast to the Chinese HSR system, none of these documented railway systems present any characteristic scale. In a wider context of PTSs, exponential or power-law-like distributions are also very common in aviation, maritime and urban street networks. The scale-free property denoted by such degree distributions has been speculated as a general feature of many PTSs. In accordance with this general pattern, these PTSs are thought to have similar evolutionary dynamics, referred to as ‘preferential attachment’ [11, 46]. This mechanism postulates that a station with a higher connection with other stations is more likely to be connected to newly constructed stations in the growing network (the so-called ‘rich-get-richer’ phenomenon). Our study provides an important exception to this general pattern, suggesting that a rather different mechanism may underpin the Chinese HSR network. Although a deeper understanding of its evolutionary dynamics would require a systematic accumulation of time-series data (this is beyond the scope and data availability of this study), a possible explanation could rest on the top-down vs. bottom-up nature of network formation. Scale-free property seems to have been primarily found in networks that have been constructed in a bottom-up, or loosely, self-organized manner. In this process, the joining of a new link is driven by the strong potential of passenger flow between the node pair, more succinctly, at places where passengers have strong needs to travel between stations. In contrast, the rapid development of the Chinese HSR system is largely a result of top-down planning [47]. Instead of developing in a gradually growing manner (in which preferential attachment can happen), many stations and routes were planned and constructed almost simultaneously. According to the national-level master plan of the Chinese HSR system [47], the core routes are referred to as those that serve as the backbone of the system, forming a spatial plan of ‘*Eight Horizontal and Eight Vertical Trunk Lines*’ to connect regional core cities, thereby producing a ‘radiative effect’ throughout the whole country. The secondary routes are intended to facilitate regional-level transportation by connecting core cities with their surrounding secondary cities. Placement of the partitioned high- vs. low-degree nodes on the map provides a clear picture showing that these nodes correspond well with the stations on the core versus secondary routes, respectively (Fig 1d).

Betweenness centrality and closeness centrality also exhibit bimodal-like distributions. Although the second mode in the frequency distribution of betweenness centrality is less clear than that of the other two metrics, we can also observe the distinction between core versus secondary routes by separating the low- and high-centrality nodes (Fig 1). Putting together the three different metrics yields a convergent pattern which shows that the stations along the core routes have higher degrees. Also, they tend to be necessary intermediate nodes located on the shortest paths of node pairs across the entire network (indicated by high betweenness centrality), and are relatively close in distance with the other nodes (indicated by high closeness centrality).

**Fig 3:**
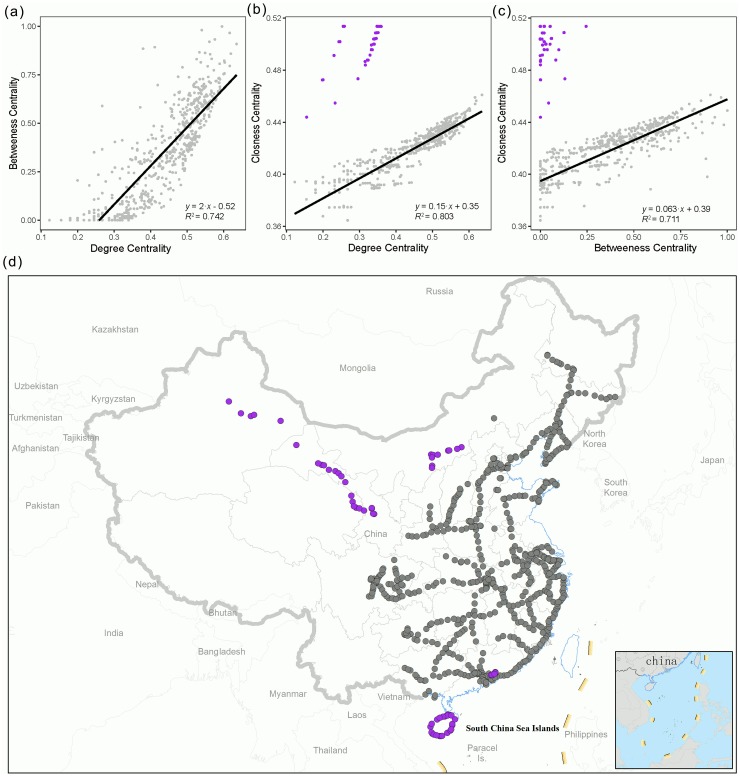
Pairwise relationships between the three network metrics (a-c). The purple-point groups deviating from the main cluster are identical in (**b)** and **(c)**, representing stations (purple dots) that are isolated from the main body (gray dots) of the network **(d)**. Univariate linear regression with the ordinary least square method shows that all pairwise relationships are significant at the level of *P*<0.001. The purple dots as deviants are excluded from the regression analysis in **(b)** and **(c)**. All metrics are log-transformed and then normalized to 0–1.

A visual comparison between the mapped stations with high-centrality versus low-centrality of network metrics may give an intuitive impression that they present a similar structure (Fig 1d–1f). Indeed, this intuition can be confirmed using a quantitative analysis based on the Venn diagram (i.e., both the low- and high-centrality parts consist of very similar station sets for different metrics; Fig 2), as well as by examining the strong correlations between the metrics (Fig 3). The inconsistency is mostly caused by closeness centrality (Fig 2), and the ‘deviants’ (purple points in Fig 3) are mostly isolated parts of the major body of the HSR system (e.g., those in remote western China and on Hainan Island).

Ultimately, our results provide clear evidence that the Chinese HSR system is shaped with a hierarchical structure, which is consistently reflected by the three network metrics. Our findings could have useful implications for future studies on this strikingly unique PTS. An implication can be linked to the consequences of socioeconomic development, since there has been evidence that the HSR has a positive effect on the economy at the city level [31,48]. If this effect can be indeed pronounced countrywide, a corollary is that the hierarchical structure of HSR would cascade to the entire social system, potentially influencing distribution (e.g., rank-size distribution of cities), inequality, and other aspects of society. However, the classical measures of network structure used in this study are rather simple, so more advanced analyses of network structure are needed in future studies.

For a more complete understanding of the Chinese HSR system, our findings based on statistical patterns of network metrics can provide an important addition to the previous work that addressed its spatial features [32]. In that work [32], a spatial view helps to answer the questions including: Is the realized spatial structure of the HSR network consistent with the master plan? Where are the stations/cities that have the most important network functions, in terms of high centrality? Are there any modular sub-networks, and if so, where are they? The present study used the same dataset but occupies an essentially different niche from [32]. Here we show that a non-spatial, topology-based view can reveal the hierarchical structure as a more fundamental, holistic nature of network. In this sense, being complementary with each other, both spatial and non-spatial views would contribute to our understanding of complex networks.

The possibility that the pattern found in this study is a transient state that arises during system formation cannot be ruled out. This raises an interesting question. Would the HSR system eventually approach scale-free structure, or would the hierarchical feature persist over time? Fortunately, perhaps there is no need to wait too long to gather sufficient time-series data to better understand the dynamics and to assess the socio-geographical influences of the largest HSR system in the world due to the increased availability of real-time open big data.

## Supporting information

S1 File The Chinese high-speed rail network data used in this study. The data file can be opened by the open software Gephi and any text editor such as Notepad.
(NET)
